# Delivering an Electronic Health Record Based Educational Intervention Promoting Peri-Operative Non-Pharmacological Pain Care as Part of a Randomized Controlled Trial: Mixed Method Evaluation of Inpatient Nurses’ Perspectives

**DOI:** 10.2196/70332

**Published:** 2025-07-17

**Authors:** Sarah A Minteer, Cindy Tofthagen, Kathy Sheffield, Susanne Cutshall, Susan Launder, Jane Hein, Mary McGough, Christy M Audeh, Jon C Tilburt, Andrea L Cheville

**Affiliations:** 1 Mayo Clinic, 200 First St SW, Rochester, MN, 55905, United States, 1 507-422-6907; 2 Mayo Clinic, Jacksonville, FL, United States; 3 Mayo Clinic, Phoenix, AZ, United States

**Keywords:** non-pharmacological pain care, clinical decision support, electronic health record, nurse perspectives, pain management, patient education

## Abstract

**Background:**

Best practice guidelines recommend educating surgical patients about non-pharmacological pain care (NPPC) techniques that can be used in addition to pain medication for perioperative pain management, given the risks for opioid misuse following surgery. As part of the parent non-pharmacologic options in postoperative hospital-based and rehabilitation pain management (NOHARM) clinical trial, we implemented the Healing After Surgery initiative, which leveraged the Epic electronic health record (EHR) to provide patients with education on NPPC techniques perioperatively. We disseminated educational materials directly to patients via the EHR patient portal and prompted patients to select the techniques they were most interested in using, which auto-populated the EHR so that their care team could view their preferences. We also built clinical decision support elements in the EHR to prompt and support inpatient nurses in providing patients with education and reinforcement for using their preferred NPPC techniques. Print materials, a website, a DVD, videos on hospital televisions, a toll-free number, and Zoom-based group calls provided additional education on NPPC techniques.

**Objective:**

This study evaluated nurses’ perceptions of barriers and facilitators to implementing the EHR-based Healing After Surgery initiative.

**Methods:**

We invited inpatient nursing leaders and bedside nurses to participate in a semistructured interview. Inpatient nursing leaders were invited to complete a brief survey that asked them to rate their agreement with 7 items using a numeric rating scale (1=not at all, 10=a great deal).

**Results:**

Interview findings from 29 nurses revealed: (1) nurses gravitated towards providing NPPC techniques they were familiar with, (2) the initiative was patient-centric with opportunities to better engage patients, and (3) nurses experienced challenges implementing and prioritizing the intervention in the inpatient setting due to competing demands in a pandemic and postpandemic environment. Interviews revealed mixed effectiveness of implementation strategies. We received survey responses from 47 nursing leaders who indicated that their staff knew about the Healing After Surgery initiative (mean=7.53, SD=1.77) and what they were expected to do (mean=7, SD=1.88). They thought the Healing After Surgery initiative supported patients’ pain management needs (mean=6.76, SD=2.24), endorsed it as a priority (mean=7.02, SD=2.56), and encouraged staff to support it (mean=5.98, SD=2.78). They indicated staff experienced some burden supporting the initiative (mean=3.93, SD=2.47), but supported some variation of the initiative continuing once the parent trial ended (mean=7.72, SD=2.62).

**Conclusions:**

Nurses understood the intervention’s benefit but struggled to implement unfamiliar NPPC techniques and prioritize the initiative due to other clinical demands. Additional implementation strategies may be needed to better engage patients and facilitate intervention delivery.

## Introduction

Patients undergoing a surgical procedure in the United States are often prescribed, and at times overprescribed, opioids for managing peri-operative pain [1,2]. Recent guidelines suggest a conservative approach to opioid prescribing and dosing [3] given the risk of improper opioid use post-operatively [4,5]. Other research suggests that limiting the duration of post-operative use may be more important than limiting dosage [6].

As the risks of addiction have become increasingly publicized, patients may be concerned about the risks associated with taking opioids [7]. A survey of patients who had undergone surgery found 30% endorsed concerns about developing an opioid addiction [8]. As a result, patients may desire information about appropriate medication use and how and when to discontinue use [9]. Preoperative education in this area has been found to be effective at reducing patients’ opioid consumption [10-12].

When encouraging patients to limit opioid use, offering alternative strategies for managing postoperative pain becomes increasingly important [13]. In addition to nonopioid pain medications, nonpharmacologic pain management techniques may offer another means of pain management [14-17]. Patients may also benefit from having their care teams introduce nonpharmacological pain care (NPPC) during their postoperative recovery, as patients may be unlikely to incorporate these techniques on their own [18]. However, these techniques may not be routinely provided to patients [8,18].

Some nurses may not feel equipped to advise patients on the full spectrum of nonpharmacologic options [7]. Deficits in knowledge about and training in nonpharmacologic techniques and lack of time may prevent nurses from using them [19,20]. Oncology nurses, for example, were more likely to use nonpharmacologic techniques with patients if they thought those strategies were effective and they had institutional support (eg, time, equipment, knowledge, and peer, colleague, and administration support) for doing so [21]. Critical care nurses used techniques they possessed knowledge and training in, personally used, and perceived as legitimate and beneficial [19]. Importantly, nurses’ beliefs of legitimacy and training may vary greatly for different non-pharmacological techniques [19]. Education on pain management in nursing school is sparse, and specifics of what to cover are determined by programs [22]. Nursing curricula may provide nurses with deficient knowledge of pain management, including NPPC [23], but providing specific training on NPPC can increase nurses’ intentions to incorporate these techniques into their practice [24].

The Healing After Surgery initiative, evaluated in the nonpharmacologic options in postoperative hospital-based and rehabilitation pain management (NOHARM) clinically-embedded, pragmatic clinical trial [25], was designed to provide patients with education and support for incorporating NPPC techniques into their individualized perioperative pain management plans. Inpatient nurses played a large role in supporting the Healing After Surgery initiative by providing patients with educational resources on their preferred NPPC techniques (including directing them to interactive resources), delivering NPPC as feasible, and discussing NPPC as part of the discharge conversation. We received strong support and endorsement for nursing’s involvement from enterprise nursing leadership. This mixed methods analysis explores inpatient nursing leaders’ and bedside nurses’ perceptions of barriers and facilitators to implementing the Healing After Surgery initiative. The goal was to understand aspects of the intervention and implementation strategies that promoted implementation and challenges nurses encountered with implementation to inform similar future efforts implementing large-scale EHR-based educational initiatives that rely heavily on nurses to support implementation. This manuscript was prepared in accordance with the consolidated criteria for reporting qualitative research (COREQ) guidelines [26].

## Methods

### Study Design

For this study, we conducted individual, semistructured interviews with nursing leaders and bedside nurses to obtain detailed feedback about facilitators and barriers to implementing the Healing After Surgery initiative in inpatient settings. We also conducted a brief, investigator-developed web-based survey, which was completed by inpatient nursing leaders to evaluate barriers and facilitators to the implementation of NPPC as part of the Healing After Surgery initiative.

### The NOHARM Clinical Trial and Healing After Surgery Initiative

The NOHARM pragmatic clinical trial [25] used a stepped-wedge cohort cluster-randomized design. The trial was conducted in 31 surgical practices across six sites belonging to the Mayo Clinic Enterprise: Rochester, Minnesota; Phoenix, Arizona; Jacksonville, Florida; Mankato, Minnesota; Eau Claire, Wisconsin; and La Crosse, Wisconsin. Practices comprised the following surgical specialties: lung, cardiac, gynecology, obstetrics, transplant, colorectal, orthopedics. Practices were randomly assigned to clusters defined by site and surgical practice to initiate the Healing After Surgery initiative during one of five sequences (steps in the wedge) at seven-month intervals. The control comparator condition was usual care. At the start of the trial, all surgical practices underwent a seven-month control interval. The first group of surgical practices began delivering the intervention in March 2021, the second group began delivering the intervention in October 2021, the third group began delivering the intervention in May 2022, the fourth group began delivering the intervention in December 2022, and the fifth group began delivering the intervention in July 2023. Patient outcomes of the NOHARM trial comparing patients undergoing the intervention to those who received usual care and qualitative research on patients’ perceptions of their experiences with the intervention will both be reported elsewhere. The present manuscript focuses on nurses’ experiences delivering the Healing After Surgery initiative.

The Healing After Surgery initiative introduced surgical patients to 13 evidence-based NPPC techniques via a Healing After Surgery guide that was automatically sent to their EHR patient portals when they were identified as having a qualifying surgery within the next 30 days. The 13 NPPC techniques included movement techniques (walking, yoga, tai chi), physical techniques (transcutaneous electrical nerve stimulation (TENS), massage, acupressure, cold or heat), and relaxation techniques (meditation, guided imagery, aromatherapy, relaxing breathing, muscle relaxation, and music listening). Patients were encouraged to select up to three NPPC techniques of greatest appeal using the guide, which created discrete structured EHR data elements, which drove clinical decision support (CDS) including provider interfaces. Patients’ NPPC choices were viewable in discipline-specific EHR workflow. These interfaces allowed inpatient care teams (eg, inpatient nurses, physical and occupational therapists) to view and support patients’ preferences. If a patient had not indicated their NPPC preferences or their preferred techniques were not appropriate, inpatient nurses were instructed to share educational resources on the different NPPC techniques and encourage the patient to choose (other) NPPC techniques and enter the patient’s preferences into the EHR. Inpatient nurses were encouraged to provide patients with educational materials on their preferred NPPC techniques and deliver NPPC for pain relief as feasible and compatible with existing nursing workflows. Healing After Surgery videos on hospital televisions, print materials stocked on inpatient floors, and information about NPPC techniques that auto-populated the patient’s discharge summary supported inpatient nurses’ educational efforts.

Epic CDS tools included best practice advisories for the provision of preferred NPPC for elevated pain, education points for preferred NPPC, a clickable Healing After Surgery banner at the top of the patient’s chart to indicate Healing After Surgery initiative participation, and task reminders to educate the patient on NPPC [25] were built into the EHR to support inpatient nurses in delivering the initiative. There was also a designated pager nurses could call with questions. Nurses were encouraged to direct patients to interactive resources staffed by the research team (eg, Healing After Surgery patient toll-free number, Healing After Surgery Zoom-based group calls held three times a week) and a Healing After Surgery website, if they did not have the time or knowledge for more in-depth discussions of the patient’s preferred NPPC techniques.

Inpatient nurses were onboarded to this program via an online educational module. The study’s implementation team worked with nursing unit leaders to determine the best way to provide additional education and support to nurses. The implementation team was comprised of a diverse group of study team members including implementation researchers, physicians, nurses (eg, nurse scientist, clinical nurse specialist, and nurse manager), physical therapists, a physical therapy assistant, an occupational therapist, and study coordinators who cumulatively possessed implementation research and clinical expertise. Examples of additional education and support provided by the implementation team included presenting at staff meetings or professional development days or holding drop-in sessions with lunch or snacks for staff. We also offered in-person and online TENS training to teach nurses how to use the TENS machines we provided to their units. Nurses who started their job after this initial onboarding had access to the online educational module, but this was not required education for all new employees.

Upon implementation, members of the implementation team intermittently stopped by units to provide additional support. The implementation team also used secure messaging (available through the EHR) to offer nurses one-on-one support and to encourage them to ask questions. However, these supports were only available to day shift nurses. Our team also held wellness events for staff about midway through the trial, in which staff could stop by a room where members of our team offered some of the NPPC techniques.

### Interview Design

Interviews were semistructured, using an interview guide developed by the study team, including those with clinical and research expertise. Interview questions were informed by the Consolidated Framework for Implementation Research (CFIR) 2.0 [27], which is a well-known implementation framework consisting of 5 domains (innovation, outer setting, inner setting, individuals, and implementation process) that contain related constructs. This framework can help elucidate what helped or hindered implementation. Interview questions were developed before, and independently of, survey development.

### Survey Design

The brief survey, housed in the web-based platform, RedCap, had 2 questions that asked about participants’ general characteristics (eg, work site and nursing role). In total, 7 additional items asked participants to indicate the extent to which staff knew about the initiative and what was expected of them to support the initiative; the extent to which the initiative helped support patients’ pain management needs; how burdensome it was for staff to support the initiative; how big of a priority it was for their staff to support the initiative; how much they continued to encourage and remind staff to support the initiative; and to what extent they would support a variation of the initiative becoming part of standard care after the trial ended. These 7 questions were rated on a 10-point numeric rating scale, and participants were provided the anchors, 1=not at all, 10=a great deal. Participants could also select "unable to assess" as a response. Survey items were generated by members of the study team (including nursing professionals, a researcher, and physicians), whose experience helping implement the intervention informed key questions for nursing leadership to assess how implementation was going. The survey was reviewed by a member of the study team with expertise in survey research but was not pilot tested.

### Sample

Nursing leaders (eg, supervisors, administrators, interim managers, clinical nurse specialists, and nursing education specialists) from inpatient floors that regularly cared for Healing After Surgery patients from each of the 31 surgical practices included in the NOHARM trial were invited to participate in a brief survey and interview. Inpatient nurses who regularly delivered bedside care to Healing After Surgery patients from the 31 surgical practices were also eligible to participate in interviews.

### Recruitment

We had originally planned to invite nursing leaders to participate in an interview approximately one year after their unit had begun delivering the Healing After Surgery initiative to give their unit time to adopt the intervention. This meant nursing leaders would be recruited at different timepoints depending on which of the 5 timepoints their surgical practice had been randomized to begin delivering the intervention as part of the parent trial. Our goal was to recruit 2‐3 key inpatient nursing stakeholders from all 31 surgical practices. (Sometimes inpatient units cared for patients from multiple surgical practices or patients from a single surgical practice were cared for across multiple surgical floors with different nursing leadership and staff).

We emailed inpatient nursing leaders from the first group of surgical practices to begin delivering the Healing After Surgery initiative approximately 15 months later and invited them to participate in a 30-minute interview. The email informed nursing leaders they could also identify a representative for their unit (eg, charge nurse) who may be interested in participating. We emailed nursing leaders from the second group of surgical practices to begin delivering the Healing After Surgery initiative approximately one year after their units had begun intervention delivery. We also included information about the opportunity for bedside nurses to participate in an interview in study newsletters sent to inpatient nursing leaders from the first 2 groups of practices to begin delivering the intervention. However, recruitment was challenging, likely due to the COVID-19 pandemic environment and high rates of nursing leadership and staff turnover. Thus, we modified our recruitment email to more clearly state we were seeking 1 nursing leader and 1 bedside nurse from each surgical practice to participate in an interview and recontacted nursing leaders from the first 2 practice groups. Bedsides nurses were instructed to contact the study team if interested in participating in a research interview. Including both a nursing leader and bedside nurse from each practice increased the likelihood of achieving broad representation from the 31 surgical practices. In addition, because bedside nurses were directly involved in delivering the Healing After Surgery initiative to patients, their perspectives on facilitators and barriers to implementation complement the perspectives of nursing leaders, who oversaw implementation of the Healing After Surgery initiative on their unit but were not directly involved in its delivery to patients.

The third group of practices to begin delivering the intervention received the updated recruitment email approximately 1 year after they began delivering the intervention. However, because of ongoing challenges with recruitment and high rates of leadership and staff turnover, we recruited nurses from the fourth group of practices approximately six months after they began delivering the initiative and recruited nurses from the fifth group of practices approximately three months after they began delivering the initiative. These shorter intervals between beginning delivery of the initiative and recruitment were intended to minimize the likelihood of turnover while providing enough time for practices to adopt the initiative. For surgical practices that experienced leadership turnover and for which we had not met our recruitment goal, we invited new leaders to participate in interviews.

Ultimately, we invited 113 nursing leaders and bedside nurses to participate in an interview. We contacted bedside nurses about participating in an interview after they emailed us to express interest. The total number of nurses eligible to participate in an interview therefore exceeded 113, but it was impossible to determine how many additional bedside nurses may have become aware of the opportunity to participate via newsletter or heard about it from their leadership but never contacted us. A maximum of three recruitment attempts were made.

To offer nursing leaders a less time-consuming way to provide their feedback on the initiative and allow us to evaluate facilitators and barriers to implementation, we invited 78 nursing supervisors, administrators, managers, nursing education specialists, and clinical nurse specialists that represented the 31 surgical practices to complete a brief survey between July and December 2023. Recruitment was conducted via in-person outreach, email, or telephone. Nursing leaders from the fifth group of practices to begin delivering the initiative were not recruited to complete the survey until at least approximately three months since they had begun delivering the intervention, to ensure adequate exposure to the initiative. A maximum of 3 recruitment attempts were made.

### Procedure

Print surveys were delivered with pre-addressed envelopes. Surveys returned through the health care enterprise’s internal mail system were entered into Qualtrics by the study coordinator. Those who preferred to complete the survey online were emailed a link to the Qualtrics survey. A study coordinator entered responses into Qualtrics for any participants who preferred to complete the survey via phone.

For the interviews, two female, PhD-level researchers (SAM and CT) conducted the interviews. SAM is a research associate, and CT is a nurse scientist. Both had extensive interview and qualitative research experience from other research projects. SAM has also attended a workshop on rapid qualitative analysis and a workshop on qualitative analysis in implementation science. SAM and CT helped implement the Healing After Surgery initiative, so some participants were familiar with them from study implementation activities. Participants were informed at the start of the interview that the interviews were being conducted for the purpose of understanding what went well and what did not in terms of implementation. Interviews were conducted via telephone or a web-based video platform, Zoom, and only attended by the interviewer and participant. All interviews were recorded, and the audio was transcribed verbatim.

### Ethical Considerations

For the survey, completion of the survey was taken as assent to participate, and those who did not wish to participate could choose not to. Participants provided verbal consent for participating in an interview. Respondents received $35 remuneration for completing a survey and $50 remuneration for completing an interview. Survey data were reported in aggregate. Exemplary quotes were attributed to a participant ID number assigned by the study team to protect participants’ identities. The Mayo Clinic Institutional Review Board approved all procedures, #21‐007898.

### Analytic Strategy

We calculated descriptive statistics for responses gathered from the leadership surveys. Numeric responses were averaged across survey respondents as a whole and by site.

For the nurse interviews, we used Rapid Analysis [28,29] to summarize interview transcripts. Rapid analysis is a technique that can be used when the results are needed to modify implementation strategies of evidence-based interventions [30]. Consistent with the rapid analytic approach which follows a deductive process, SM developed a Summary Template containing domain labels based on the interview guide questions to facilitate summarizing and organizing key information from interview transcripts. For the first three interviews, SAM, CT, and KS (a Senior Clinical Research Coordinator) independently completed summaries by summarizing information from the interview transcripts as bullet points under the appropriate domain. They met to discuss their summaries after each of these three interviews to establish consensus for completion. Remaining interview transcripts were summarized by either SAM, CT, or KS, independently. SAM reviewed all completed summaries, making minor edits, before uploading them into Nvivo software by Lumivero [31]. In Nvivo, the text for each summary was coded to the corresponding domain on the summary template, and the text for each domain was queried. SAM and CT created analytic memos for queried domains, identifying key themes and how they fit within CFIR 2.0. SAM and CT met to discuss key themes and their correspondence with CFIR 2.0 constructs and arrived at consensus via discussion. CT and SAM further consolidated key themes within CFIR 2.0 via independent preparation of memos and discussion.

## Results

### Qualitative Findings

We interviewed 29 of the 113 inpatient nurses (26% response rate) invited to participate between June 2022 and November 2023. Of those interviewed, 13 were in leadership roles and 16 were in clinical, direct patient care roles; 27 were female and 2 were male. Participants worked in Rochester, Minnesota (n=10); Phoenix, Arizona (n=7); Jacksonville, Florida (n=4); Eau Claire, Wisconsin (n=3); La Crosse, Wisconsin (n=2); and Mankato, Minnesota (n=3). They cared for the following types of surgical patients: colorectal (n=3), obstetrics (cesarean section; n=5), gynecological (n=3), cardiac ICU (n=3), pulmonary and thoracic (n=1), transplant (n=3), orthopedic (n=5), cardiac and lung (n=4), gynecological and orthopedic (n=1), and colorectal and gynecological (n=1). Interviews lasted 32 minutes on average (minimum=20 minutes, maximum=47 minutes).

Interviews revealed the following three themes related to barriers and facilitators to the implementation of the Healing After Surgery initiative: (1) nurses tended to gravitate towards NPPC techniques they were familiar with, (2) the initiative was patient-centric, but opportunities remained to better engage patients via increased care team communication, and (3) nurses experienced challenges implementing and prioritizing the intervention in the inpatient settings due to competing demands experienced in the pandemic and postpandemic environment. CFIR 2.0 constructs that correspond to each theme are shown in parentheses.

#### Nurses Tended to Gravitate Toward NPPC Techniques They Were Familiar With (Compatibility, Innovation Deliverers–Capability, and Available Resources–Materials & Equipment)

The initiative’s focus on pain management incorporating non-pharmacologic techniques broadly aligned with the Health Care System’s current approach to patient care. However, nurses varied in their receptiveness towards (eg, thoughts about appropriateness of inpatient use) and familiarity with different NPPC techniques and perceived the availability of resources to vary. More specifically, walking, application of ice, music, and relaxation techniques were available in current practice, while techniques such as yoga, tai chi, and acupressure were not. There were mixed impressions regarding the compatibility of TENS (transcutaneous electrical nerve stimulation), aromatherapy, and massage with nursing practice. Some nurses were concerned they may be asked to deliver techniques for which they felt they lacked the training or resources (eg, extra time or special equipment) to deliver. As one participant described it,

“it’s just that it’s time, massage is time, TENS unit is time…and unfortunately, I feel like that has been very short lately.”

(P18, lines 531‐532).

There was a tendency for nurses to discuss NPPC techniques with patients that they were personally familiar with and avoid discussing techniques they were less familiar with or lacked resources to provide. One nurse stated,

“I don’t know the first thing about tai chi, or…how we would be able to implement that in the inpatient setting at least. Maybe once they go home, they could utilize that more.”

(P1, lines 38‐40).

However, some units were resourceful with figuring out how to get the supplies they needed, such as ordering essential oils to support aromatherapy.

#### Initiative Viewed as Patient-Centric, but Opportunities Remained to Better Engage Patients via Increased Care Team Communication (Recipient-Centeredness, Assessing Needs–Innovation Recipients, and Engaging Innovation Recipients)

Offering options for nonpharmacologic pain management empowered patients by offering pain management solutions that extend beyond the scope of medications, allowing patients to be active participants in their pain management plan. One nurse shared,

“I feel like this is just another part of nursing, and we should have, to be honest, probably been doing this all along... I think patients are very receptive to it, very thankful for it, and I feel like it makes a significant difference when it’s done”.

(P13, lines 776‐782).

NPPC was viewed as particularly helpful for patients who were reticent to take opioids and for use in conjunction with medications for individuals experiencing severe pain. Patients tended to gravitate toward more familiar techniques such as walking, application of cold or heat, or relaxed breathing; however, there were medical situations and priorities that made engaging in NPPC impractical. For instance, persons with serious, acute needs, those who had emergency surgery, new mothers who had a cesarean section, and patients who were intubated were not good candidates for the intervention.

Nurses also identified missed opportunities to increase patient engagement and felt strongly that conversations about NPPC use should be initiated before surgery. They emphasized that patients would likely be more engaged if they had heard about the Healing After Surgery program from their providers in advance, as an example:

I don’t know if this is something that they’re currently doing, but I do think that having providers share the same message….really helps…and not just in those times where… nursing can’t necessarily do it because of timing… but… have that as part of their normal kind of workflow or discussion points with patients.

(P21, lines 363‐371).

While it fell within the scope of nursing practice to support NPPC, there were suggestions to engage others in delivery, including physicians, virtual RNs, and nurse technicians. Nurse technicians are members of the health care team that function under the direct supervision of a registered nurse, performing basic patient care tasks such as vital signs and assistance with activities of daily living.

#### Challenges Implementing and Prioritizing the Intervention in the Inpatient Setting (Physical Infrastructure, Relative Priority, Critical Incidents, Work Infrastructure, Individuals–Mid-Level Leaders–Capability, Motivation, Deliverer-Centeredness, and Mission Alignment)

The intervention was introduced during the COVID-19 pandemic, and although the elective surgical patient population targeted by the intervention did not typically have a COVID-19 infection, the pandemic indirectly impacted the delivery of the intervention due to staff burnout and changes to work infrastructure. Nurses noted bed shortages, higher patient to staff ratios, and caring for higher acuity patients as barriers to delivering the intervention and NPPC. Patients also had shorter hospital stays than in years prior. One nurse shared:

just five years ago, a knee or a hip would be in the hospital for probably a minimum of three days. Now…we got about one and a half to two days maybe. And that’s from the time they check in down at the desk.... So when I say that 1.5, we’re really getting about 24 hours with the patient to do this education…that’s probably our biggest challenge, but that’s across the board...

(P7; 501‐521).

The COVID-19 pandemic also created an environment of increased staff and leadership turnover. New leaders were unfamiliar with what training on the intervention staff had received. With leadership turnover, the intervention got deprioritized, and many leaders did not do much to convey support for the initiative, particularly after the initial roll-out:

…we’ve…had a lot of leadership turnover in the last couple of years... and we’re… in the limbo of interim managers and all of that right now. So it’s… hard because I feel like we’ve missed a lot of that solidarity of the same person and that support just ’cause everyone’s trying to pick up the pieces and just get us through until the next manager takes over… right when they rolled out, they were super supportive…since then, we’ve…been in this limbo

(P24; 385‐400).

Many saw this initiative as something extra and deprioritized it when faced with short staffing and other tasks. One nurse stated:

…the way that they staff us for… our floors …you don’t have extra time to do…the education that you want to be able to do…these extra interventions that you wanna do. You have enough time to do …the bare minimum stuff…if it’s a really good day, and everything is going perfectly, sometimes you get those extra moments

(P18; 175‐180).

For new nurses joining units, the priority was on developing their basic nursing skills. One nurse shared,

… when your staff… is stretched and they’re new you have to go back to the basics to make sure that everyone has the basic skills…and NOHARMs is… very important, but it’s not a basic skill. So it doesn’t go to the top of my list [laughter].

(P5; 346‐354).

In this context, some nurses tended to look towards medications first for faster results.

A few nurses thought that this initiative should be prioritized, and it was a reasonable ask. A few leaders also communicated the importance of the initiative or how it aligned with broader goals for patient care and opioid reduction. One nurse manager stated,

…we have to realize that we are… hurting people more than helping them with our heavy-handed narcotic passing. And so just knowing that importance and expressing that to the team, I felt like it is a high-priority item…you’re always gonna be able to make an excuse about something being a low-priority problem, you know?...—as a nurse manager, it can be overwhelming to decide what you fit where, but it doesn’t mean that you can’t fit it.

(P2; lines 483‐501).

Leaders who conveyed support did so via existing communication channels (eg, email and huddle), and some went out of their way to make sure staff were informed (eg, posting fliers in the breakroom). Interviewees suggested getting support from additional councils and quality and practice committees might have enhanced support.

#### Mixed Effectiveness of Implementation Strategies (Design, Access to Knowledge & Information, Implementation Leads, Engaging Innovation Deliverers, and Tailoring Strategies)

Nurses reflected on several evidence-based implementation strategies used by the research team or units themselves to facilitate implementation of the intervention, which had varied success. Implementation strategies were part of the Expert Recommendations for Implementing Change (ERIC) list of strategies [32]. These are summarized in Table 1.

**Table 1.** Nurses’ reflections on different ERIC^a^ implementation strategies used.

Description of implementation strategies	Exemplary quotes
ERIC implementation strategies: Develop educational materials, Distribute educational materials, Conduct educational meetings	
The study team developed a robust plan for educating nurses about the intervention and how to support its delivery, including assigning a MyLearning module via the institution’s web-based educational platform for staff and offering to present at staff meetings or hold drop-in sessions. However, nurses thought that they had received a general overview on the intervention, but additional training and follow-up were needed. They shared that MyLearning modules are sometimes forgotten because of the amount of modules they are assigned to watch and that drop-in sessions and presentations at staff meetings likely do not capture all staff. Interviews also highlighted the lack of a systematic process for training new hires and nurses who “float” to different floors despite high rates of staff turnover and float nursing coverage on floors.	“…I have done so many MyLearning on it. [Laughter] don’t remember if I—was there one? I don’t even know...” P8; lines 227‐230 “Because of the turnover on the floor that the initial education was just before we got there, a lot of us. I think that might be a… barrier.” P17; 113‐119
ERIC implementation strategy: Identify and prepare champions	
The study team did not explicitly ask units to identify champions or super-users, and interviews revealed this rarely occurred organically. Nurses discussed the high rate of staff turnover and the number of new staff as interfering with recruiting champions for initiatives in general. However, some nurses identified those in leadership roles on their unit as super users (eg, clinical nurse specialist, nursing educational specialist, team leads, charge nurse). A couple also shared that their unit had a champion at one point, who then left.	“I think everyone’s pretty good at like trying to bounce ideas off each other and things like that. And I think everyone’s pretty open to… trying alternatives… but …I’m not aware of somebody… it’s kind of hard right now with the staff turnover. We have so many young staff members. So, we’re having a hard time … filling positions on unit council, and filling positions on… all the different things like that. But we do have… a falls champion, and a pressure ulcer champion… we have two wellness champions. But it’s not always super easy to get those positions filled.*” P18; 566‐586* “…we…chose to make our team lead groups, so we’ve got 12 team leads, 6 that’re core, that’s their primary job, and 6 that’re relief, which means they fill in as needed our super-user group, just because there’s always one of them on either day shift or night shift. And they can be that…go-to person if somebody has questions.” *P15; 423‐428*
ERIC implementation strategy: Remind clinicians	
Nurses generally viewed the clinical decision support (CDS) elements in the electronic health record (EHR) as helpful and compatible with nursing workflow. Some nurses were not aware of some of the CDS, which suggests they were not overly intrusive.	“seemed very straightforward and similar to what we… had previously done with other educational elements.” P3; 386‐390
Many respondents reported that they or their leaders shared information in meetings or huddles, checked in with staff during rounding, and put information about the intervention in emails/newsletters.	“reminders… emails, huddles…the little… daily interactions to say…. ‘Hey, have you guys been doing this?’ Or, ‘What’s been working? What’s not?’ ….I think they’re pretty good about… reminding us… that we’re still a part of that and… our goal is to.. do this… it’s become a part of my practice that, even if I have a patient that’s not a NOHARM patient, I’m still… talking about those same points with them.” P19; 315‐323
ERIC implementation strategy: Provide local technical assistance	
The study team used the EHR-based messaging system, secure chat, to offer to answer nurses’ questions about intervention delivery and Epic EHR documentation. Some nurses thought this was helpful and unintrusive. However, others thought messages sent by the study team got lost with other messages they received, were of lower priority, and may have been confusing to float nurses, who did not regularly work on that inpatient floor and may have been unfamiliar with the initiative.	“…through secure chat… is very beneficial ’cause… I do know that there have been times where you guys have noticed that maybe we weren’t following through with the plan the way we should, and there’s that, ‘Hey, please remember this patient wants to do X, Y, and Z.’… it’s a gentle way of letting us know that, “Hey, there’s this other thing out there,” without being…. waiting by the door, waiting for that nurse to come out and then… accosting them in the hallway, so to speak.” P7; 460‐473
ERIC implementation strategy: Conduct educational outreach visits	
Members of the implementation team stopped by inpatient floors to support staff, which was perceived to be helpful. However, night shift nurses were not well engaged.	“I think definitely having someone on the unit to be there to… answer questions, to actually physically show you the chart is definitely more helpful than a MyLearning. [Laughter] I think that you just hafta click through ’cause it’s another required education.” P12; 266‐270 “…I personally work a lot of night shifts… so I haven’t seen anybody from No Harm come around besides the one-day shift when they rolled this out and gave us information…” P24; 568‐576
ERIC implementation strategy: Audit and feedback	
Some nursing leaders shared report data provided by the research team on how their unit was doing with intervention delivery in emails/newsletters.	“… when you give us updates of where we’re at as far as meeting the percentages of… patients that have done their selection…I’m trying to remember if it also talks about the discharge teaching…we share those results in our newsletters. We do a weekly newsletter, so when… you guys send us that, then we… share that in our newsletter that we send out to the staff.” P5; 390‐405
ERIC implementation strategy: Change physical structure and equipment	
Few nurses reported tailoring implementation of the intervention but modifications that were reported were mostly related to changing the location of Healing After Surgery-related resources to make them more accessible.	“…we started putting on each individual pod—so it’s more accessible—... aromatherapy kits. Where it was in one central location behind the team lead’s, it is now available to every pod in our med room... As well as printouts right there of the list, so it’s easily accessible rather than trying to dig through a drawer or look it up on the intranet to try to print something out. It’s just right there and available...” P19; 523‐532

a ERIC: Expert Recommendations for Implementing Change [32].

### Survey Findings

We received survey responses from 47 of the 78 (60% response rate) nursing leaders invited. See Table 2 for respondents’ work site and professional role. Overall, leaders thought their staff knew what the Healing After Surgery initiative was (mean=7.53, SD=1.77), and that their staff knew what they were expected to do to support the initiative (mean=7, SD=1.88). The initiative was perceived to support patients’ pain management needs (mean=6.76, SD=2.24), but added some burden to staff (mean=3.93, SD=2.47). Leaders endorsed the Healing After Surgery initiative as a priority (mean=7.02, SD=2.56) and rated their ongoing encouragement of staff to support the initiative slightly lower (mean=5.98, SD=2.78). Generally, nursing leaders supported the Healing After Surgery program becoming part of standard care once the clinical trial ended (mean=7.72, SD=2.62).

Respondents from the hospital located in Rochester, Minnesota, rated burden from this initiative the highest (mean=5, SD=2.68), and their ongoing encouragement for staff to support this initiative (mean=4.73, SD=2.24) and support for this initiative to continue (mean=6.09, SD=2.63) the lowest of all sites. Respondents from the hospital in Phoenix, Arizona, rated the ability of the initiative to help them better meet patients’ pain management needs (mean=8.25, SD=1.60) and their interest in having the initiative continue in some form (mean=8.75, SD=1.76) the highest of all sites. Respondents from the hospital in La Crosse, Wisconsin, rated staff’s knowledge of what the initiative was (mean=9.33, SD=1.15) and staff’s knowledge of what was personally expected (mean=8.6, SD=2.31) of them highest of all sites. See Table 3 for nursing leaders’ responses by site (and to view the survey questions).

**Table 2.** Survey participants by role and site.

Site	Nurse administrator	Nurse manager	Interim nurse manager	Clinical nurse specialist	Nursing education specialist	Other	Total
Rochester, Minnesota	1	7	—^a^	2	1	—	11
Phoenix, Arizona	—	4	1	1	3	3	12
Jacksonville, Florida	—	7	—	1	1	—	9
Eau Claire, Wisconsin	—	4	—	1	2	—	7
La Crosse, Wisconsin	—	2	—	—	1	—	3
Mankato, Minnesota	—	2	—	2	1	—	5

a Not applicable.

**Table 3.** Mean (SD) responses to individual survey items by site.

Question	Rochester, MN	Phoenix, AZ	Jacksonville, FL	Eau Claire, WI	La Crosse, WI	Mankato, MN
To the best of your knowledge, to what extent do your staff know what the Healing After Surgery initiative is?	6.55 (1.51)	8.5 (1.51)	7.22 (2.17)	7.43 (1.51)	9.33 (1.15)	7 (1.41)
To the best of your knowledge, to what extent do your staff know what they are personally expected to do to support the Healing After Surgery initiative?	6.36 (1.50)	8.17 (1.80)	5.89 (1.90)	7.29 (1.38)	8.67 (2.31)	6.2 (1.48)
To what extent has the Healing After Surgery initiative helped you and your staff better support patients’ pain management needs?	6.36 (2.62)	8.25 (1.60)	6.44 (2.13)	5.71 (1.89)	5 (2.83)^a^	6.75 (2.22)^a^
How burdensome has it been for your staff to support the Healing After Surgery initiative?	5 (2.68)	3.18 (2.44)^a^	3.89 (2.85)	3.14 (1.46)	4 (3.61)	4.4 (1.95)
How big of a priority is it to you that your staff support the Healing After Surgery initiative?	5.91 (2.34)	7.75 (2.09)	8.56 (1.67)	5.57 (3.60)	7 (2.65)	7 (2.71)^a^
Outside of early implementation efforts, how much have you continued to encourage and remind staff to support the Healing After Surgery initiative? (eg, email reminders, discussions, etc.)	4.73 (2.24)	5.33 (3.26)	6.78 (2.82)	7.43 (2.51)	7.33 (2.52)	6 (2.55)
To what extent would you support a variation of the Healing After Surgery Program becoming part of standard care once the trial ends?	6.09 (2.63)	8.75 (1.76)	8.44 (2.01)	7.43 (2.51)	7.67 (2.52)	8 (0.71)

a One participant from the site selected “unable to assess” so their response is not included in the mean.

## Discussion

### Principal Findings

This study describes nurses’ perceptions of NPPC and implementation of the Healing After Surgery initiative across diverse surgical practices within a single healthcare system spanning multiple geographic locations. The Healing After Surgery initiative was developed as a low-touch intervention designed to provide patients with peri-operative education and support for using NPPC for perioperative pain management. This educational initiative was intended to cover the perioperative period and include all members of the surgical care team, addressing inadequacies in education on pain management and opioid safety and setting of pain expectations previously reported [33]. During the pre-operative period, patients were assigned an educational Healing After Surgery guide upon surgical scheduling. Ambulatory nurses and preoperative evaluation clinic staff encouraged patients to complete the guide and offered additional educational materials during preoperative visits. Surgeons were also encouraged to voice their support of the initiative to patients. Inpatient nurses were designated as having a key role in intervention delivery by providing patients with education on their preferred NPPC techniques and delivering them as feasible during the postoperative inpatient stay.

To minimize the burden of intervention delivery on inpatient nurses, we developed multimodal educational resources (eg, print resources, website, DVD, and videos available on hospital televisions) designed to support patients in self-administration of NPPC. For example, print materials and a brief video guided patients through finding pressure points and applying pressure to facilitate patients’ use of acupressure. Videos on the website guided patients through tai chi and gentle yoga movements geared towards a surgical patient population. Moreover, training provided to nurses instructed them to provide patients with these resources (eg, give patients print materials stocked on their floor, navigate to the NPPC educational videos on the hospital television). Nurses were also told that they did not need to be experts in the NPPC techniques. Nurses could discuss NPPC techniques as their knowledge and time allowed and refer patients needing more support to the Zoom-based group calls and toll-free number.

Interview and survey responses revealed that the initiative was thought to be patient-centric and help meet patients’ pain management needs. Nursing leaders were supportive of NPPC practice integration and its continuation notwithstanding some burdens in supporting the program. However, challenges to implementing the Healing After Surgery initiative identified via the interviews included nurses’ lack of familiarity with some of the NPPC modalities and lack of time to educate patients on NPPC due to other aspects of patient care that took priority. Further, some of the implementation strategies employed could be modified to better support implementation.

Four of the ERIC implementation strategies utilized to support nursing implementation of the Healing After Surgery initiative included developing educational materials, distributing educational materials, conducting educational meetings, and conducting outreach visits [32] with the goal of training nurses how to deliver the intervention. However, these strategies (eg, an assigned web-based educational module, presentations at staff meetings, “drop-in sessions,” and at-the-elbow support) may have failed to adequately reach all nurses due to high rates of staff turnover, nursing float coverage (eg, nurses taking shifts on a unit that was not their usual unit), or nurses not being scheduled to work during these supports. Moreover, these efforts made nurses aware of available patient educational resources and CDS developed as a reminder of what to do and to facilitate documentation. Education on the NPPC techniques was a lesser focus. We thought nurses would be familiar with many of the techniques because pain management, including self-management strategies, is an essential competency for nurses [34-36]. We also thought nurses would be comfortable directing patients to educational materials or the toll-free number and Zoom-based group calls for NPPC techniques while realizing they may have lacked the time or knowledge to educate patients on the techniques. However, most nurses gravitated to those NPPC modalities already integrated into their practice (eg, walking, application of ice) and avoided discussing techniques they were not sure how to use (eg, tai chi and acupressure). Although nurses are likely introduced to NPPC during their training and include basic techniques such as application of cold or heat and walking into their practice, they may benefit from additional training in the use of techniques that they are less familiar with and not comfortable discussing with patients. Similarly, a survey of critical care nurses found that roughly 80% and 59% reported no training or knowledge in tai chi and acupressure, respectively [19]. Furthermore, only roughly 1% of those who reported no training or knowledge in these techniques utilized them as part of their practice [19]. Anecdotal conversation during the implementation of the Healing After Surgery initiative suggested that giving patients materials without being able to discuss them may run counter to nursing practice. This might explain why some nurses avoided mentioning NPPC techniques they were not comfortable with altogether.

To normalize NPPC use in hospitals, it may be important to bolster nurse knowledge and familiarity with relevant, evidence-based pain modalities via additional training [19,37-41], and nursing curriculums should consider integrating more information about NPPC [37,42]. Education could take the form of brief in-services [37], patient stories, hands-on learning, e-learning, lunch-and-learns, education days, and speaking with colleagues [7]. However, it is important to ensure that night shift nurses have the same access to these opportunities as day shift nurses. We only provided implementation support (eg, presentations at huddles and staff meetings, pager support, at-the-elbow support on inpatient floors) during daytime hours. Some practices may have overcome this by having charge nurses or team leads be super users (eg, clinical champions), but this was driven by units themselves and not routinely adopted.

Additional education on NPPC may also help address concerns about the appropriateness and feasibility of some of these techniques that persisted despite emphasizing in the nursing training that our educational materials were created for a surgical patient population. Delivering techniques such as yoga or tai chi to patients in the hospital was perceived to be impractical. Further, although we had not initially planned on educating critical care nurses on how to support the Healing After Surgery initiative, some of the included surgical practices routinely admitted patients to the intensive care unit (ICU) following their procedure, and in cases of high hospital census, patients could spend more time there. Thus, in partnership with ICU nursing leadership, we determined that critical care nurses may be able to introduce NPPC to patients depending on the patient’s stage of recovery. However, past studies have described patient-related barriers to pain management in intensive care units [43], and whether the Healing After Surgery initiative was appropriate for patients on critical care units varied depending on their status.

Some of the interviewees also described competing patient care priorities and suggested that inpatient units are not ideal for teaching NPPC to patients. For instance, some nurses perceived NPPC as an “extra” and relied on pharmacological pain management as a fast and effective method of alleviating pain. Our findings suggest that current inpatient systems and processes within our hospital enterprise may not allow enough time for nurses to readily deliver NPPC for pain management despite strong endorsement from the enterprise and participating surgical departments for implementing the HAS initiative. Literature has noted that nurse staffing ratios may impede pain management [38,44,45] and NPPC use [40,42], and competing demands on nurses’ time may also impede NPPC use [39,41,42,44,45]. The implementation strategies we utilized to prompt, support, and motivate nurses to provide patients with NPPC education (eg, CDS clinician reminders, local technical assistance via EHR-based messaging, and audit and feedback data on completion of NPPC selections and NPPC education) may have limited effectiveness if time and competing priorities remain important barriers. One helpful implementation strategy used by some units was changing the physical location of some Healing After Surgery-related resources to make them more accessible to staff (eg, less time and effort to retrieve materials). However, small modifications like this may be insufficient to overcome time constraints imposed by higher patient to staff ratios and higher acuity patients, exacerbated by the pandemic. The healthcare enterprise in which this initiative was delivered may consider more explicitly voicing prioritization of NPPC provision (and how this should be prioritized compared to other nursing responsibilities) and ensure nurses are given the necessary time [39], if the goal is for this to become a routine part of peri-operative pain management.

Interviews also suggested that patients could benefit from the patient’s surgeon also discussing NPPC with patients. We did encourage surgeons to voice their support of the HAS initiative to patients, but it is unclear to what extent this occurred. Moreover, there may be an opportunity for advanced practice providers (APPs) to voice their support of NPPC as well, although we minimally engaged them as part of the initiative. A prior survey of APPs conducted at one of the hospitals in which the present study took place found that APPs held positive beliefs about NPPC, but discussion about the benefits and risks of NPPC occurred inconsistently [46]. However, if surgeons and APPs consistently voice support for NPPC, this may help create an organizational culture in which discussion about NPPC as part of peri-operative pain management is normalized and expected.

### Strengths and Limitations

This study gathered interview data from inpatient nursing leaders and bedside nurses and survey data from inpatient nursing leaders on the barriers and facilitators to implementing a program designed to provide patient education and support for use of NPPC for peri-operative pain management. CFIR 2.0 guided the qualitative data collection and interpretation, adding to its theoretical rigor. Furthermore, our findings add to the literature by including inpatient nurses from diverse surgical practices and sites that varied in academic status, rurality, and size. Some site differences did emerge, perhaps due to competing priorities, adequacy of staffing, nursing leaders’ own biases, patient volumes, and hospital culture. The variation in the support at different locations may also reflect the overall complexity with size of each facility, surgical volumes, culture, resources, and complexity of care during the time the study was conducted during the COVID-19 pandemic. However, only nurse leaders were offered participation in the survey, and the number of respondents from each site varied, so it is unclear to what extent survey findings reflected the opinions of each site. It is also possible that the opinions and perspectives of the nursing leaders may or may not adequately reflect the opinions and perspectives of all involved bedside nurses. Further, although our response rate was 60% for the survey and 26% for the interviews, we make no definitive claim of representativeness because all hospital sites belong to the same health care enterprise, instead focusing on the range of issues identified in this context. Nonetheless, our findings, particularly from the interviews, were fairly balanced in terms of identifying facilitators and barriers to implementation, suggesting our results were not skewed in one direction.

The study began during the COVID-19 pandemic, which provided a unique implementation context that might have influenced our findings. However, our interviews suggested that the elective surgical nature of this patient population did not seriously impact their experience. Instead, COVID-19 more indirectly impacted inpatient surgical units in terms of staffing and higher rates of staff turnover. It may also be difficult to compare findings from our research to that of other studies that have used validated implementation measures because survey items were generated by the members of the research team and not taken from a validated scale. Nonetheless, the items have face validity and achieved our objective of understanding nursing leaders’ perceptions of facilitators and barriers to the implementation of the Healing After Surgery initiative.

### Conclusions and Future Directions

Our findings highlight important lessons learned during the implementation of a novel EHR-facilitated initiative into inpatient nursing practices that incorporate additional education and provision of non-pharmacologic options for managing pain. This trial provides feedback on the use of technology to align care to meet guidelines for comprehensive pain management initiated prior to surgery and during recovery at home. Our findings highlight a need for earlier education and increased availability of resources to support the use of NPPC. Moreover, our findings suggest challenges and opportunities for better engagement of inpatient nurses for any practice change at the inpatient nurse level, given the anticipated persistence of similar complexity of care and short lengths of stay. Our findings also highlight a challenge inherent to large-scale pragmatic trials that require engaging and training a large number of intervention providers to deliver an intervention with fidelity. Future efforts may explore other ways of strategically augmenting implementation resources to effectively engage care teams. There are opportunities to enhance nurses’ role in pain management with educational support for NPPC in academic programs, through orientation support, virtual nursing roles, and ongoing professional development support.

## Abbreviations

CDS: clinical decision support
CFIR: Consolidated Framework for Implementation Research
EHR: electronic health record
ERIC: Expert Recommendations for Implementing Change
NOHARM: Nonpharmacologic Options in postoperative Hospital-based and Rehabilitation pain Management
NPPC: nonpharmacological pain care
TENS: transcutaneous electrical nerve stimulation

## References

[1] El Moheb M, Mokhtari A, Han K (2020). Pain or no pain, we will give you opioids: relationship between number of opioid pills prescribed and severity of pain after operation in US vs Non-US patients. J Am Coll Surg.

[2] Thiels CA, Anderson SS, Ubl DS (2017). Wide variation and overprescription of opioids after elective surgery. Ann Surg.

[3] Dowell D, Ragan KR, Jones CM, Baldwin GT, Chou R (2022). CDC clinical practice guideline for prescribing opioids for pain - United States, 2022. MMWR Recomm Rep.

[4] Barth RJ, Waljee JF (2020). Classification of opioid dependence, abuse, or overdose in opioid-naive patients as a “never event”. JAMA Surg.

[5] Gil JA, Gunaseelan V, DeFroda SF, Brummett CM, Bedi A, Waljee JF (2019). Risk of prolonged opioid use among opioid-naïve patients after common shoulder arthroscopy procedures. Am J Sports Med.

[6] Brat GA, Agniel D, Beam A (2018). Postsurgical prescriptions for opioid naive patients and association with overdose and misuse: retrospective cohort study. BMJ.

[7] Leegaard M, Watt-Watson J, McGillion M, Costello J, Elgie-Watson J, Partridge K (2011). Nurses’ educational needs for pain management of post-cardiac surgery patients: a qualitative study. J Cardiovasc Nurs.

[8] Gan TJ, Habib AS, Miller TE, White W, Apfelbaum JL (2014). Incidence, patient satisfaction, and perceptions of post-surgical pain: results from a US national survey. Curr Med Res Opin.

[9] Kennedy D, Wainwright A, Pereira L (2017). A qualitative study of patient education needs for hip and knee replacement. BMC Musculoskelet Disord.

[10] Egan KG, De Souza M, Muenks E, Nazir N, Korentager R (2020). Opioid consumption following breast surgery decreases with a brief educational intervention: a randomized, controlled trial. Ann Surg Oncol.

[11] Lee BH, Wu CL (2020). Educating patients regarding pain management and safe opioid use after surgery: a narrative review. Anesth Analg.

[12] Rucinski K, Cook JL (2020). Effects of preoperative opioid education on postoperative opioid use and pain management in orthopaedics: A systematic review. J Orthop.

[13] Chou R, Gordon DB, de Leon-Casasola OA (2016). Management of postoperative pain: a clinical practice guideline from the American pain society, the American society of regional anesthesia and pain medicine, and the American society of anesthesiologists’ committee on regional anesthesia, executive committee, and administrative council. J Pain.

[14] Tick H, Nielsen A, Pelletier KR (2018). Evidence-based nonpharmacologic strategies for comprehensive pain care: the consortium pain task force white paper. Explore (NY).

[15] Sezen CB, Akboga SA, Celik A, Kalafat CE, Tastepe AI (2017). Transcutaneous electrical nerve stimulation effect on postoperative complications. Asian Cardiovasc Thorac Ann.

[16] Fan M, Chen Z (2020). A systematic review of non-pharmacological interventions used for pain relief after orthopedic surgical procedures. Exp Ther Med.

[17] Good M, Anderson GC, Stanton-Hicks M, Grass JA, Makii M (2002). Relaxation and music reduce pain after gynecologic surgery. Pain Manag Nurs.

[18] Keast M, Hutchinson AF, Khaw D, McDonall J (2022). Impact of pain on postoperative recovery and participation in care following knee arthroplasty surgery: a qualitative descriptive study. Pain Manag Nurs.

[19] Tracy MF, Lindquist R, Watanuki S (2003). Nurse attitudes towards the use of complementary and alternative therapies in critical care. Heart Lung.

[20] Kidanemariam BY, Elsholz T, Simel LL, Tesfamariam EH, Andemeskel YM (2020). Utilization of non-pharmacological methods and the perceived barriers for adult postoperative pain management by the nurses at selected national hospitals in Asmara, Eritrea. BMC Nurs.

[21] Kwekkeboom KL, Bumpus M, Wanta B, Serlin RC (2008). Oncology nurses’ use of nondrug pain interventions in practice. J Pain Symptom Manage.

[22] Compton P, Blacher S (2020). Nursing education in the midst of the opioid crisis. Pain Manag Nurs.

[23] Latchman J (2014). Improving pain management at the nursing education level: evaluating knowledge and attitudes. J Adv Pract Oncol.

[24] Murphy-Smith MT, Samawi Z, Abbott P (2024). Teaching strategies for nonpharmacological pain management to nursing students. Pain Manag Nurs.

[25] Redmond S, Mayo Clinic NRT, Tilburt J, Cheville A (2022). Non-pharmacological options in postoperative hospital-based and rehabilitation pain management (NOHARM): protocol for a stepped-wedge cluster-randomized pragmatic clinical trial. Pain Ther.

[26] Tong A, Sainsbury P, Craig J (2007). Consolidated criteria for reporting qualitative research (COREQ): a 32-item checklist for interviews and focus groups. Int J Qual Health Care.

[27] Damschroder LJ, Reardon CM, Widerquist MAO, Lowery J (2022). The updated consolidated framework for implementation research based on user feedback. Implement Sci.

[28] Hamilton A (2020). Rapid qualitative analysis: updates/developments VA HSR&D National Cyberseminar.

[29] Hamilton A (2013). VA HSR&D National Cyberseminar Series: Spotlight on Women’s Health.

[30] Hamilton AB, Finley EP (2019). Qualitative methods in implementation research: an introduction. Psychiatry Res.

[31] (2024). Nvivo. version version 14. www.lumivero.com.

[32] Powell BJ, Waltz TJ, Chinman MJ (2015). A refined compilation of implementation strategies: results from the Expert Recommendations for Implementing Change (ERIC) project. Implement Sci.

[33] Nallani R, Fox CC, Sykes KJ (2021). Pain management and education for ambulatory surgery: a qualitative study of perioperative nurses. J Surg Res.

[34] Pain management core competencies for prelicensure clinical education: an interprofessional consensus. American Association of Colleges of Nursing.

[35] Fishman SM, Young HM, Lucas Arwood E (2013). Core competencies for pain management: results of an interprofessional consensus summit. Pain Med.

[36] Herr K, Marie BS, Gordon DB (2015). An interprofessional consensus of core competencies for prelicensure education in pain management: curriculum application for nursing. J Nurs Educ.

[37] Gumus K, Musuroglu S, Karaman Ozlu Z, Tasci O (2020). Determining the use of nonpharmacologic methods by surgical nurses for postoperative pain management and the influencing professional factors: a multicenter study. J Perianesth Nurs.

[38] Ayaz NP, Sherman DW (2022). Understanding attitudes, social norms, and behaviors of a cohort of post-operative nurses related to pain and pain management. Healthcare (Basel).

[39] He HG, Jahja R, Lee TL (2010). Nurses’ use of non-pharmacological methods in children’s postoperative pain management: educational intervention study. J Adv Nurs.

[40] He HG, Pölkki T, Vehviläinen-Julkunen K, Pietilä AM (2005). Chinese nurses’ use of non-pharmacological methods in children’s postoperative pain relief. J Adv Nurs.

[41] Pölkki T, Laukkala H, Vehviläinen-Julkunen K, Pietilä AM (2003). Factors influencing nurses’ use of nonpharmacological pain alleviation methods in paediatric patients. Scand J Caring Sci.

[42] Alrimali AM, Al-Hamad NM, Almazeani FH, Alharbi MD (2023). Nonpharmacological pain management practices among nurses working in multiple centers in Saudi Arabia: A cross-sectional study. Journal of Integrative Nursing.

[43] Rababa M, Al-Sabbah S, Hayajneh AA (2021). Nurses’ perceived barriers to and facilitators of pain assessment and management in critical care patients: a systematic review. J Pain Res.

[44] Mędrzycka-Dąbrowska W, Dąbrowski S, Basiński A (2015). Problems and barriers in ensuring effective acute and post-operative pain management--an international perspective. Adv Clin Exp Med.

[45] Elcigil A, Maltepe H, Eşrefgil G, Mutafoglu K (2011). Nurses’ perceived barriers to assessment and managementof pain in a university hospital. J Pediatr Hematol Oncol.

[46] Bauer BA, Townsend KM, Cutshall SM (2020). Advanced practice providers’ knowledge, attitudes, and utilization of complementary and integrative medicine at an academic medical center. Altern Ther Health Med.

